# Contraction Integral Equation for Three-Dimensional Electromagnetic Inverse Scattering Problems

**DOI:** 10.3390/jimaging5020027

**Published:** 2019-02-08

**Authors:** Yu Zhong, Kuiwen Xu

**Affiliations:** 1 Institute of High Performance Computing, Agency for Science, Technology and Research (A*STAR), Singapore 138632, Singapore; 2 Key Lab of RF Circuits and Systems of Ministry of Education, Hangzhou Dianzi University, Hangzhou 310018, China

**Keywords:** inverse scattering, nonlinear problem, contraction integral equation for inversion (CIE-I), imaging

## Abstract

Inverse scattering problems (ISPs) stand at the center of many important imaging applications, such as geophysical explorations, industrial non-destructive testing, bio-medical imaging, etc. Recently, a new type of contraction integral equation for inversion (CIE-I) has been proposed to tackle the two-dimensional electromagnetic ISPs, in which the usually employed Lippmann–Schwinger integral equation (LSIE) is transformed into a new form with a modified medium contrast via a contraction mapping. With the CIE-I, the multiple scattering effects, i.e., the physical reason for the nonlinearity in the ISPs, is substantially suppressed in estimating the modified contrast, without compromising physical modeling. In this paper, we firstly propose to implement this new CIE-I for the three-dimensional ISPs. With the help of the FFT type twofold subspace-based optimization method (TSOM), when handling the highly nonlinear problems with strong scatterers, those with higher contrast and/or larger dimensions (in terms of wavelengths), the performance of the inversions with CIE-I is much better than the ones with the LSIE, wherein inversions usually converge to local minima that may be far away from the solution. In addition, when handling the moderate scatterers (those the LSIE modeling can still handle), the convergence speed of the proposed method with CIE-I is much faster than the one with the LSIE. Secondly, we propose to relax the contraction mapping condition, i.e., different contraction mappings are used in updating contrast sources and contrast, and we find that the convergence can be further accelerated. Several numerical tests illustrate the aforementioned interests.

## 1. Introduction

**Figure 1:**
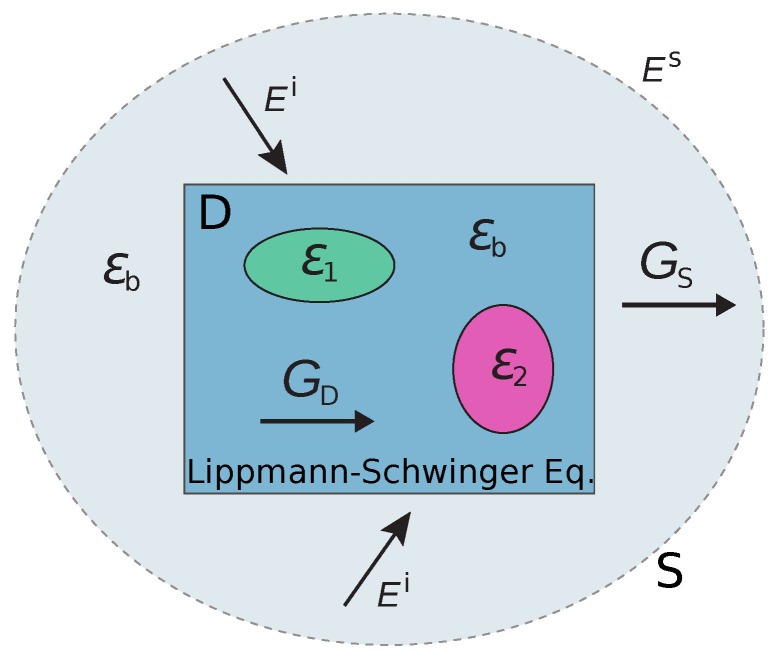
Schematics of inverse scattering problems.

Inverse scattering problems (ISPs) in electromagnetics and acoustics are of great interest in industries due to important imaging applications in various areas, such as geophysical survey, non-destructive testing, ground-penetrating radar, bio-medical imaging, etc., as solving the ISPs provides rich information about the unknown targets, such as locations, shapes, and material distributions within some structures [1,2,3,4]. For instance, microwave imaging has been used to inspect the abnormalities in human bodies like bleeding in the head and tumours in breasts. On the other hand, they are also quite important due to the representative difficulties in solving a large group of inverse problems concerning waves and fields, and thus researchers in mathematics, physics, and engineering societies have devoted great efforts to improving efficiencies and accuracies of the numerical solvers [5]. As shown in Figure 1, solving ISPs is to determine the unknown scatterers within a domain, when the domain is illuminated by several different incidences and we can measure the scattered fields outside the domain (usually contaminated by noise) for each incidence. It is well known that the main difficulties in such ISPs are their two intrinsic properties, i.e., ill-posedness and nonlinearity [1]. As most of the practical problems are three-dimensional (3-D) ones, which require much more computational resources than those in two-dimensional cases, they are often more difficult to solve due to the data deficiency (measurement aperture usually covers a small solid angle), and therefore also stronger ill-posedness and nonlinearity. Great efforts have been paid to tackle these demanding problems, for instance [6,7,8,9].

Methods for solving ISPs can be catalogued into two types of optimization approaches: deterministic and stochastic. The deterministic type of the inversion methods has been developed for decades, such as the contrast source inversion (CSI) method [6,10,11], the Born iterative method and distorted Born iterative method [12,13], the level set method [14,15], the subspace-based optimization method (SOM) [8,16,17,18], etc. The second type, the stochastic type of the inversion methods, usually employs a group of initial guesses and uses the stochastic optimization scheme to minimize the objective function, such as the genetic algorithm and the evolutionary optimization. The stochastic type methods increase the possibilities of finding the global minimum rather than being trapped in a local minimum as the deterministic optimization techniques [19,20]. Both techniques have also been applied to solve 3-D inverse scattering problems [6,21,22,23,24]. Other than the quantitative methods mentioned above, some qualitative methods, such as the linear sampling method [25] and other methods, like [26], are proposed to retrieve the geometric supports of the unknown scatterers.

To tackle the nonlinearity, the major difficulty in efficiently solving the ISPs, different types of integral equations have been proposed, such as in [27,28,29]. In particular, motivated by the contraction integral equation (CIE) in solving the direct scattering problems with highly conductive background media [30], a new type of contraction integral equation for inversion (CIE-I) has been proposed [29] to tackle the two-dimensional highly nonlinear ISPs by transforming the usually employed Lippmann–Schwinger integral equation (LSIE) into a new form with a modified medium contrast via a contraction mapping. With the CIE-I, the global multiple scattering effects (MSE) are substantially suppressed in estimating the modified contrast in the CSI type methods. With the FFT type twofold SOM regularization scheme [8], the inversion solver with CIE-I is capable of effectively alleviating the nonlinearity of the two-dimensional ISPs, especially those highly nonlinear ones with strong scatterers with large contrast and/or large dimensions (in terms of wavelengths). In this paper, this new CIE-I will be implemented in tackling the 3-D ISPs.

In summary, the contributions of the paper include:

1. The CIE-I is firstly implemented to tackle the computationally costly full 3-D ISPs, to address the highly nonlinear 3-D ISPs, and to accelerate the convergence of the inversions.
2. A relaxed type of inversion scheme based on CIE-I is proposed, with different auxiliary parameters β (the parameter in CIE-I to control the portion of MSE in estimating the contrast) in updating the contrast sources and in updating the contrast. This means to further accelerate the convergence of the inversions.
3. Several numerical tests are provided with details, for the sake of further algorithmic studies.

After the Introduction, the proposed 3-D inversion method is detailed in Section 2. In Section 3, three numerical examples are given to validate the proposed method. Finally, conclusions are drawn.

## 2. Inversion with CIE-I

In this paper, the domains of interest (DoI) are chosen to be rectangular cuboid in order to implement the conjugate gradient fast Fourier transform (CG-FFT) scheme and apply the Fourier basis in TSOM. For the convenience of reading, in this paper, we denote the one-dimensional tensor as $\bar{a}$, two-dimensional tensor as $\bar{\bar{a}}$, three-dimensional tensor as $\hat{a}$, and four-dimensional tensor as $\hat{\hat{a}}$. Unless otherwise specified, the subscript of the tensors denotes the index of the element, such as ${\bar{\bar{a}}}_{m,n}$ denotes the element in $\bar{\bar{a}}$ with index {m,n}. We use bold symbols to denote vectorial physical quantities, such as the positions r and the electric fields E in 3-D cases.

### 2.1. 3-D Modeling

In 3-D cases, there are $N_{i}$ incident waves from different angles impinging onto the rectangular cuboid DoI D ($D\subset R^{3}$, the background 3-D homogeneous medium with permittivity $\epsilon_{0}$ and permeability $\mu_{0}$), where nonmagnetic scatterers are located, and these incident waves are expressed as $E_{l}^{\mathrm{inc}}\left(r\right)$, $l=1,2,\cdots ,N_{i}$, r∈D. For each incidence, the scattered fields are collected by $N_{r}$ receivers located at $r_{j}^{\prime }$, $j=1,2,\cdots ,N_{r}$. With all such information, including every incident field inside the domain of interest and the corresponding scattered fields at the positions of all detectors, we aim at determining the dielectric profile ϵ(r), r∈D.

The scattering models are governed by the following electric field volume integral equation based on the well-known Lippmann–Schwinger integral equation (LSIE). For the $l^{\mathrm{th}}$ incidence, the field equation in the domain D is expressed as

(1) $$I_{l}\left(r\right)=\chi \left(r\right)E_{l}^{\mathrm{inc}}\left(r\right)+\chi \left(r\right)\left(G_{D}^{3D}I_{l}\right)\left(r\right),$$

where $\chi \left(r\right)=\left(\epsilon_{r}\left(r\right)-1\right)$ is the contrast, $\epsilon_{r}\left(r\right)$, $I_{l}\left(r\right)=\chi \left(r\right)E_{l}^{\mathrm{tot}}\left(r\right)$ and $E_{l}^{\mathrm{inc}}\left(r\right)$ are the relative permittivity, the contrast source and the incident electric field at r, respectively, and $\left(G_{D}^{3D}I_{l}\right)\left(r\right)$ is an integral operator with the dyadic Green’s function as the integral kernel, which can be written as [8,31],

(2) $$\left(G_{D}^{3D}I_{l}\right)\left(r\right):=k_{0}^{2}\int_{D}\left(\bar{\bar{I}}+\frac{\nabla \nabla }{k_{0}^{2}}\right)\cdot g\left(r,r^{\prime }\right)I_{l}\left(r^{\prime }\right)dr^{\prime },$$

with $k_{0}=\omega \sqrt{\epsilon_{0}\mu_{0}}$ as the wave number of the background medium, and $g(r,r^{\prime })$ as the 3-D Green’s function for the background homogeneous medium. Notice that the $I_{l}\left(r\right)$ is the contrast source in this paper, while, in [8], it is the physically induced current that includes a multiplicative factor $-i\omega \epsilon_{0}$ compared to the contrast source. Otherwise, as we know, the dyadic Green’s function is composed of nine scalar elements, which represent the mapping from the three components of the contrast source to the three components of the scattered fields inside the DoI.

The nonlinearity of ISPs comes from the MSE, as shown in Equation (1). To alleviate the nonlinearity of the model, in [29], by some mathematical manipulation on Equation (1), another new-type integral equation, which is denoted as CIE-I herein, can be obtained

(3) $$\beta \left(r\right)I_{l}\left(r\right)=R\left(r\right)\beta \left(r\right)I_{l}\left(r\right)+R\left(r\right)\left[E_{l}^{\mathrm{inc}}\left(r\right)+\left(G_{D}^{3D}I_{l}\right)\left(r\right)\right],$$

where $R\left(r\right)=\left.\beta \left(r\right)\chi \left(r\right)/\left[\beta (r)\chi (r)+1\right]\right.$ is the modified contrast function, and β(r) is a chosen auxiliary parameter to control the portion of the MSE in estimating the contrast [29], which can be a constant or a variable at the different position in the DoI. For the convenience of discretizing the equation, we rewrite the dyadic Green’s function as

(4) $$\left(G_{D;uv}^{3D}I_{l;v}\right)\left(r\right):=\left\{\begin{array}{cc} k_{0}^{2}\left(1+\frac{1}{k_{0}^{2}}\frac{\partial^{2}}{\partial \iota_{u}^{2}}\right)\int_{D}g\left(r,r^{\prime }\right)I_{l;v}\left(r^{\prime }\right)dr^{\prime }, & \mathrm{if}\;u=v,\\ \frac{\partial^{2}}{\partial \iota_{u}\partial \iota_{v}}\int_{D}g\left(r,r^{\prime }\right)I_{l;v}\left(r^{\prime }\right)dr^{\prime }, & \mathrm{if}\;u\neq v, \end{array}\right.$$

where u,v=1,2,3 represent the *x*-, *y*- and *z*-components of a vector, respectively, and $\iota_{1}=x$, $\iota_{2}=y$, and $\iota_{3}=z$.

Following the conventions in [8], we can rewrite the vectorial Equation (3) into three coupled scalar equations in the discrete forms. Thus, we first discretize the rectangular domain of interest into small cuboid subdomains, whose dimensions are much smaller than the wavelength and the center of which are located at $r_{m,n,p}$, with *m*, *n*, *p* integers and $m\in [1,M_{1}]$, $n\in [1,M_{2}]$ and $p\in [1,M_{3}]$. Here, $M_{1}$, $M_{2}$, and $M_{3}$ are the total number of subdomains along *x*-, *y*-, and *z*-directions, respectively, and we let $M=M_{1}\times M_{2}\times M_{3}$ be the total number of the subdomains. With such discretization, we have

(5) $${\hat{\beta }}_{m,n,p}{\hat{I}}_{l;u;m,n,p}={\hat{R}}_{m,n,p}{\hat{\beta }}_{m,n,p}{\hat{I}}_{l;u;m,n,p}+{\hat{R}}_{m,n,p}\left[{\hat{E}}_{l;u;m,n,p}^{\mathrm{inc}}+\sum_{v=1}^{3}{\hat{G}}_{D;uv;m,n,p}^{3D}\left({\hat{I}}_{l;v}\right)\right],$$

where ${\hat{R}}_{m,n,p}=\left.{\hat{\beta }}_{m,n,p}{\hat{\chi }}_{m,n,p}/\left[{\hat{\beta }}_{m,n,p}{\hat{\chi }}_{m,n,p}+1\right]\right.$, ${\hat{\chi }}_{m,n,p}$ is the contrast at $r_{m,n,p}$, whereas ${\hat{E}}_{l;u;m,n,p}^{\mathrm{inc}}$ and ${\hat{I}}_{l;u;m,n,p}$ are the incident electric field and the induced current at $r_{m,n,p}$, respectively. Subscript u=1,2,3 denotes the *x*, *y*, and *z* components of a vector. Note that the convolution-type operators ${\hat{G}}_{D;uv}^{3D}$ are obtained via the Equation (4). For further details of the discretization of Equation (1) and the finite difference scheme to generate $\left(G_{D}^{3D}I_{l}\right)\left(r\right)$, please refer to the Appendix of [6].

Similarly, the integral operator relating the contrast sources and the scattered fields could also be expressed as the summation of the contribution from all the subdomains,

(6) $${\bar{E}}_{l}^{\mathrm{sca}}={\bar{\bar{G}}}_{S}^{3D}\cdot {\bar{I}}_{l},$$

where ${\bar{E}}_{l}^{\mathrm{sca}}={\left[{{\bar{E}}_{l;1}^{\mathrm{sca}}}^{T},{{\bar{E}}_{l;2}^{\mathrm{sca}}}^{T},{{\bar{E}}_{l;3}^{\mathrm{sca}}}^{T}\right]}^{T}$ is a $3N_{r}$ dimensional vector with ${\bar{E}}_{l;\kappa }^{\mathrm{sca}}={\left[E_{l;\kappa ;1}^{\mathrm{sca}},E_{l;\kappa ;2}^{\mathrm{sca}},\cdots ,E_{l;\kappa ;N_{r}}^{\mathrm{sca}}\right]}^{T}$ (κ=1,2,3 denotes the *x*, *y*, *z* component of the corresponding vector, respectively), ${\bar{I}}_{l}$ is a 3M dimensional vector obtained by ${\bar{I}}_{l}=\mathrm{vec}\left\{{\hat{\hat{I}}}_{l}\right\}$. In a 3-D scenario, the vectorization operation $\mathrm{vec}\left\{\cdot \right\}$ is defined to vectorize a four-dimensional tensor into a vector, i.e., if ${\bar{I}}_{l}=\mathrm{vec}\left\{{\hat{\hat{I}}}_{l}\right\}$, we have ${\bar{I}}_{l;\vartheta }={\hat{\hat{I}}}_{l;m,n,p,\kappa }$ with $\vartheta =\left(\kappa -1\right)\times M+\left(p-1\right)\times \left(M_{1}\times M_{2}\right)+\left(n-1\right)\times M_{1}+m$. In Equation (6), the scattering operator is defined as

(7) $${\bar{\bar{G}}}_{S}^{3D}=\left[\begin{array}{ccc} {\bar{\bar{G}}}_{S;11} & {\bar{\bar{G}}}_{S;12} & {\bar{\bar{G}}}_{S;13}\\ {\bar{\bar{G}}}_{S;21} & {\bar{\bar{G}}}_{S;22} & {\bar{\bar{G}}}_{S;23}\\ {\bar{\bar{G}}}_{S;31} & {\bar{\bar{G}}}_{S;32} & {\bar{\bar{G}}}_{S;33} \end{array}\right],$$

a $3N_{r}\times 3M$ matrix, with ${\bar{\bar{G}}}_{S;uv}$, a $N_{r}\times M$ matrix, the mapping from the *v* component of the induced current to the *u* component of scattered fields (the subscripts u,v=1,2,3 are not indexed for tensor elements). The explicit expression of ${\bar{\bar{G}}}_{S;uv}$ is

(8) $$\begin{array}{ll} {\bar{\bar{G}}}_{S;uv}^{3D}\left(a,b\right)= & \left\{\left(k_{0}^{2}+\frac{ik_{0}}{R_{a,b}}-\frac{1}{R_{a,b}^{2}}\right)\delta \left(u-v\right)+\right.\\ & \left.\left[{\left(r_{a}^{\prime }\right)}_{u}-{\left(r_{m,n,p}\right)}_{u}\right]\left[{\left(r_{a}^{\prime }\right)}_{v}-{\left(r_{m,n,p}\right)}_{v}\right]\left(-\frac{k_{0}^{2}}{R_{a,b}^{2}}-\frac{3ik_{0}}{R_{a,b}^{3}}+\frac{3}{R_{a,b}^{4}}\right)\right\}g\left(r_{a}^{\prime },r_{m,n,p}\right), \end{array}$$

where δ(y) is 1 when y=0 and is 0 otherwise, $R_{a,b}=\left|r_{a}^{\prime }-r_{m,n,p}\right|$ in which $b=\left(p-1\right)\times \left(M_{1}\times M_{2}\right)+\left(n-1\right)\times M_{1}+m$ with $a=1,2,\cdots ,N_{r}$, b=1,2,⋯,M, $m=1,2,\cdots ,M_{1}$, $n=1,2,\cdots ,M_{2}$, and $p=1,2,\cdots ,M_{3}$. Here, ${\left(r\right)}_{u}$ denotes the *u* component of r.

### 2.2. Objective Function for Inversions

In this subsection, we build the objective function used in the proposed inversion method, in which we will use the FFT-TSOM [8] as the regularization to stabilize the inversion, as done in [29]. Emphasize that the twofold subspace constraints have different regularization effects. The first fold, the original SOM, balances the two mismatches in the objective function, whereas the second fold, the TSOM, is the key to stabilize the inversions with CIE-I. Details are given in below.

For the original SOM part, as given in [16], with the spectral information of ${\bar{\bar{G}}}_{S}^{3D}$ (the singular value decomposition–SVD of ${\bar{\bar{G}}}_{S}^{3D}$ tells ${\bar{\bar{G}}}_{S}^{3D}\cdot {\bar{v}}_{j}^{S}=\sigma_{j}^{S}{\bar{u}}_{j}^{S}$ and the complexity of thin SVD of ${\bar{\bar{G}}}_{S}^{3D}$ is $O(27N_{r}^{2}M)$, assuming that the singular values $\sigma_{j}^{S}$ is a non-increasing sequence), the contrast sources can be decomposed into two parts, deterministic part of the contrast sources (DPCS) and ambiguous part of the contrast sources (APCS), the former being obtained as

(9) $${\bar{I}}_{l}^{d}=\sum_{j=1}^{L}\frac{{\bar{u}}_{j}^{S*}\cdot {\bar{E}}_{l}^{\mathrm{sca}}}{\sigma_{j}^{S}}{\bar{v}}_{j}^{S}={\bar{\bar{V}}}_{S}^{+}\cdot {\bar{\alpha }}_{l}^{+},$$

where ${\bar{\bar{V}}}_{S}^{+}=\left[{\bar{v}}_{1}^{S},{\bar{v}}_{2}^{S},\cdots ,{\bar{v}}_{L}^{S}\right]$, ${\bar{\alpha }}_{l}^{+}={\left[\alpha_{l;1}^{+},\alpha_{l;2}^{+},\cdots ,\alpha_{l;L}^{+}\right]}^{T}$ with $\alpha_{l;j}^{+}=\left({\bar{u}}_{j}^{S*}\cdot {\bar{E}}_{l}^{\mathrm{sca}}\right)/\sigma_{j}^{S}$, j=1,2,⋯,L, and the superscript ∗ denotes the Hermitian operation while superscript + refers to the dominant current subspace, the subspace corresponding to the dominant singular values. The value of *L* is chosen according to the noise level [16]. Later, we will see how this might work after introducing the objective function for the inversion.

For the second fold subspace constraint, according to the FFT-TSOM [8], the APCS can be written as

(10) $${\bar{I}}_{l}^{a}\left({\hat{\hat{\gamma }}}_{l}\right)={\bar{I}}_{l}^{\mathrm{tmp}}-{\bar{\bar{V}}}_{S}^{+}\cdot \left({\bar{\bar{V}}}_{S}^{+*}\cdot {\bar{I}}_{l}^{\mathrm{tmp}}\right),$$

where ${\hat{\hat{\gamma }}}_{l}=\left[{\hat{\gamma }}_{x;l};{\hat{\gamma }}_{y;l};{\hat{\gamma }}_{z;l}\right]$, ${\bar{I}}_{l}^{\mathrm{tmp}}=\left[{\bar{I}}_{x;l}^{\mathrm{tmp}};{\bar{I}}_{y;l}^{\mathrm{tmp}};{\bar{I}}_{z;l}^{\mathrm{tmp}}\right]$ with

(11) $${\bar{I}}_{u;l}^{\mathrm{tmp}}=\mathrm{vec}\left\{\mathrm{IDFT}\left\{{\hat{\gamma }}_{u;l}\right\}\right\}$$

and u=x, *y*, and *z*, IDFT is the inverse discrete Fourier transform operator, the vec{·} is the vectorization operator. Note that the inverse discrete Fourier transform (IDFT) is performed by the 3-D FFT algorithm, the computational complexity of which is $O(M\log_{2}M)$, with $M=M_{1}\times M_{2}\times M_{3}$.

By using the low-frequency Fourier components, we are able to constrain the APCS within a low-dimensional subspace. The reason [17] is to use only the contrast sources components in this subspace that is influential to the scattered fields within the DoI, such that the stability of the inversions is substantially increased. To reduce the computational costs, such a subspace can be approximately spanned by low-frequency Fourier bases [8]. Here, if we use all Fourier bases, the construction of the APCS becomes the one in the original SOM. Otherwise, we can use the low-frequency components, and set the coefficients for those high-frequency components as zeros. This can be achieved via using a mask with eight corners being 1, with size $M_{F}$ ($1\leq M_{F}\leq M_{1}/2,M_{2}/2,M_{3}/2$), and other positions being 0. The details can be found in [8].

Having expressed the contrast sources as aforementioned, it is straightforward to define the objective function. Firstly, it is natural to give the mismatch of the scattered fields by

(12) $$\Delta_{l}^{\mathrm{fie}}\left({\hat{\hat{\gamma }}}_{l}\right)={∥{\bar{\bar{G}}}_{S}\cdot {\bar{I}}_{l}^{a}+{\bar{\bar{G}}}_{S}\cdot {\bar{I}}_{l}^{d}-{\bar{E}}_{l}^{\mathrm{sca}}∥}^{2},$$

where ${\bar{I}}_{l}^{d}$ and ${\bar{I}}_{l}^{a}$ are as in Equations (9) and (10), respectively, and $∥\cdot ∥$ denotes the $L^{2}$ norm of a tensor. The current equation in Equation (5) is another key equation to satisfy. Using the APCS construction Equation (10), we define an operator as

(13) $${\left(L^{3D}\left({\hat{\hat{\gamma }}}_{l}\right)\right)}_{\kappa ;m,n,p}=\beta {\hat{I}}_{l;\kappa ;m,n,p}^{a}-\beta {\hat{R}}_{m,n,p}{\hat{I}}_{l;\kappa ;m,n,p}^{a}-{\hat{R}}_{m,n,p}\left[\sum_{v=1}^{3}{\hat{G}}_{D;\kappa v;m,n,p}^{3D}\left({\hat{I}}_{l;v}^{a}\right)\right].$$

With this definition, we could write the mismatch of Equation (5) as

(14) $$\Delta_{l}^{\mathrm{cur}}\left({\hat{\hat{\gamma }}}_{l},\hat{R}\right)={∥L^{3D}\left({\hat{\hat{\gamma }}}_{l}\right)-{\hat{\hat{\Gamma }}}_{l}^{3D}∥}^{2},$$

where ${\hat{\hat{\Gamma }}}_{l;\kappa ;m,n,p}^{3D}=-\beta {\hat{I}}_{l;\kappa ;m,n,p}^{d}+\beta {\hat{R}}_{m,n,p}{\hat{I}}_{l;\kappa ;m,n,p}^{d}+{\hat{R}}_{m,n,p}\left[\left\{\sum_{v=1}^{3}{\hat{G}}_{D;\kappa v;m,n,p}^{3D}\left({\hat{I}}_{l;v}^{d}\right)\right\}+{\hat{\hat{E}}}_{l;\kappa ;m,n,p}^{\mathrm{inc}}\right]$. Then, the objective function can be given as

(15) $$f\left({\hat{\hat{\gamma }}}_{1},{\hat{\hat{\gamma }}}_{2},\cdots ,{\hat{\hat{\gamma }}}_{N_{i}},\hat{R}\right)=\sum_{l=1}^{N_{i}}\left(\Delta_{l}^{\mathrm{fie}}/{∥{\bar{E}}_{l}^{\mathrm{sca}}∥}^{2}+\Delta_{l}^{\mathrm{cur}}/{∥{\bar{\bar{E}}}_{l}^{\mathrm{inc}}∥}^{2}\right).$$

The inversion is to minimize this objective function. As in [8], the conjugate gradient (CG) type algorithm that is used in contrast source inversion (CSI) method is adopted to minimize this nonlinear problem by alternatively updating the ${\hat{\hat{\gamma }}}_{l}$ and $\hat{R}$ at every iteration of the optimization.

### 2.3. Sketch of the Inversion Method

Following the inversion method in [8,29], we summarize it as follows:

1. Set the background medium and null APCS as the initial guesses and choose an *L* value such that the corresponding first *L* singular values of ${\bar{\bar{G}}}_{S}^{3D}$ are larger than the noise level (assuming that the noise is a white Gaussian one).
2. Set proper values for β in CIE-I modeling and proper value for $M_{F}$ to control the number of Fourier bases being used.
3. Carry out the CG type optimization algorithm to alternatively update the two types of variables, where the APCS is updated with a one-step Polak–Ribière CG scheme and the contrast is updated with the least squares method.
4. Stop the optimization if a termination condition is met, which can be a maximum number of iterations or a pre-defined relative change of APCS coefficients.
5. If the maximum number of rounds of inversion is met, go to Step 6. Otherwise, the obtained contrast and APCS will be used as the initial guesses for the next round of optimization with smaller β and larger $M_{F}$ in Step 3.
6. Output the obtained contrast.

As mentioned in [29], the first round inversion with a large β and with a low-dimensional subspace enables a fast convergence to a meaningful coarse result that could be used as an initial guess in the second round. By increasing the dimension of the APCS subspace and including more MSE in estimating the contrast, the inversion gives us a result with better resolution. Since in the second round a (supposedly) good initial guess is given, the convergence is also very fast. For some difficult problems, the inversion could be carried out with multi-round optimizations by gradually increasing the dimensions of the APCS subspace in each round while decreasing the values of β in estimating the contrast.

### 2.4. Updating Contrast and Contrast Sources with Different β

As mentioned above, there are two updates at each iteration for the two types of unknowns. In [29], the same β is used in each round of optimization for both updates. In the first round optimization in inversions, to tackle the nonlinearity, a large β is needed in updating the modified contrast, and therefore we use the same β for the update of APCS. In the subsequent rounds (instead of the first round) of optimization, for the sake of stability, a small β is used in estimating the modified contrast to cope with a high-dimensional APCS subspace, as discussed in [29]. However, there is not such a need to use a small β in updating the APCS. Consequently, in the second round of optimization, we can use a CIE-I model with larger value $\beta_{1}$ for the update of the APCS and another with smaller value $\beta_{2}$ for the update of the contrast at the same iteration. The same applies to the third round optimization, if there is such a need. The purpose of doing so is to further accelerate the convergence of these computationally burdensome 3-D inversions after the first round optimization.

**Figure 2:**
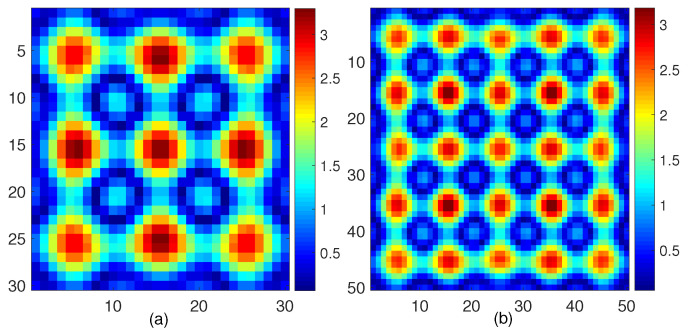
The values of $|\left(G_{D;xx}^{3D}I_{x}\right)\left(r\right)|$ in the plane x=0 when $I_{x}=1$ for a DoI with size (**a**) 3λ×3λ×3λ and (**b**) 5λ×5λ×5λ.

As for the value range for $\beta_{2}$, we carry out a similar calculation as in [29], which reflects the norm of the scattering operator that maps the contrast sources to the scattered fields within DoI. The results shown in Figure 2 indicate that, if we want to suppress the MSE in estimating the contrast when using the least squares method, we might need to choose a value for $\beta_{2}$ that is larger than 3.5, and this is the guideline for the first round optimization. For the second or subsequent rounds, as good initial guesses are provided, we can include more MSE to retrieve the fine features of the unknown scatterers.

## 3. Numerical Simulations

**Figure 3:**
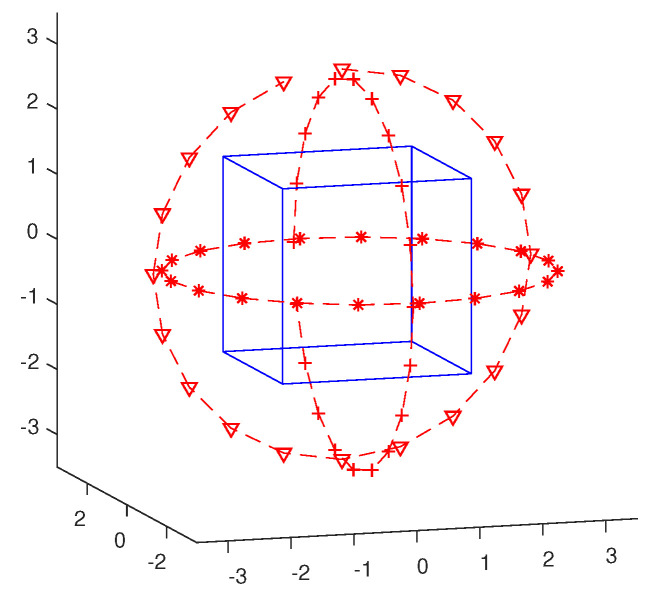
The physical setup for the numerical tests.

In this section, we will test the proposed inversion methods in three examples. We will use the same physical setup of sources and receivers for the 3-D example in [8], as shown in Figure 3. The DoI are all the same, a box with size 3λ×3λ×3λ. The box is illuminated by 60 electric dipole antennas at 300 MHz (λ=1 m in air), located at three circles (with 20 dipole antennas evenly distributed on each) with the same radius 3 m. The three circles are in x−y, y−z and x−z planes, and their centers are at (0.2, 0, –0.1), (0.1, 0, –0.15), and (–0.05, 0.1, 0), respectively. The direction of the electric dipole sources in the y−z plane are in the *x*-direction, while those in the x−z and x−y planes are in the *y*- and *z*-directions, respectively. Scattered fields are measured by 60 receivers, located at the same positions as the 60 dipole sources. We collect all three components of the fields. Consequently, we have a 60×180 synthetic data matrix. In all three tests, the synthetic data are calculated with a 60×60×60 mesh, while, in the inversions, a 30×30×30 mesh is used. All synthetic data are contaminated with additive Gaussian white noise with level of 10%.

The termination condition in each round of optimization is to reach a pre-defined relative changing rate of the APCS coefficients, given as

(16) $$\delta^{3D}=\sqrt{\frac{1}{N_{i}}\sum_{l=1}^{N_{i}}\frac{∥{\hat{\hat{\gamma }}}_{l}^{\left(h\right)}-{\hat{\hat{\gamma }}}_{l}^{\left(h-1\right)}{∥}^{2}}{∥{\hat{\hat{\gamma }}}_{l}^{\left(h-1\right)}{∥}^{2}}},$$

where ${\hat{\hat{\gamma }}}_{l}^{\left(h\right)}$ is the APCS Fourier coefficient for the $l^{\mathrm{th}}$ incidence at $h^{\mathrm{th}}$ iteration. We set $\delta^{3D}<10^{-3}$ as the termination condition.

The reconstruction results are quantitatively evaluated by the following error:

(17) $$Err=\sqrt{\frac{1}{M}\sum_{m,n,p}\frac{|{\hat{\epsilon }}_{r;m,n,p}^{\mathrm{est}}-{\hat{\epsilon }}_{r;m,n,p}^{\mathrm{tr}}{|}^{2}}{|{\hat{\epsilon }}_{r;m,n,p}^{\mathrm{tr}}{|}^{2}}}.$$

When running on a workstation with eight threads and 32 Gb RAM, every iteration of the proposed inversion method costs about 25 seconds CPU time in MATLAB, including one update on the contrast sources and one update on the contrast. Due to the independence between different incidences, we use 8 MATLAB workers to update the contrast sources in parallel.

### 3.1. Example 1

**Figure 4:**
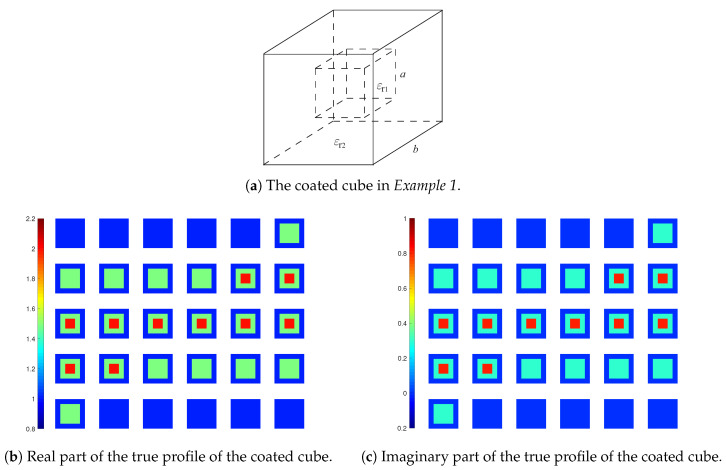
Coated cube used in *Example 1*.

**Figure 5:**
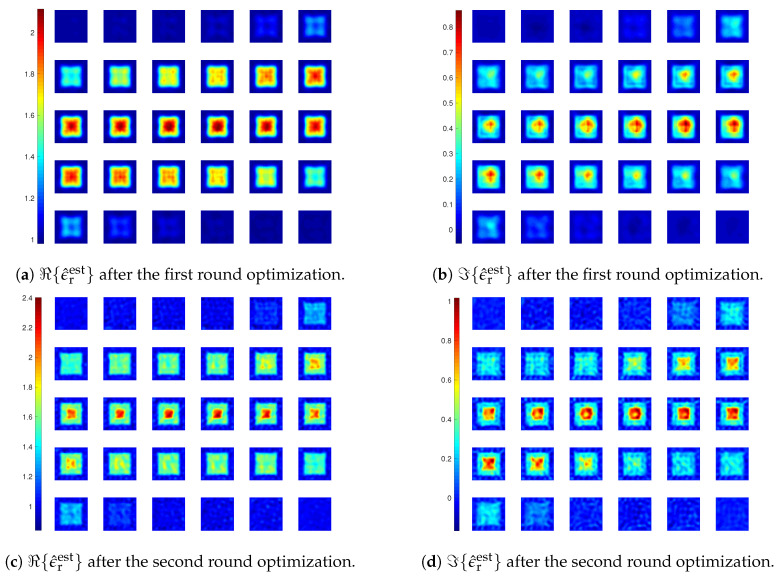
Reconstruction results of inversions with CIE-I. In the four subfigures, the 30 slices of DoI with each at $z=z_{q}$ plane, where $z_{q}$, q=1,⋯,30, are the grid points along *z*-direction, and $z_{p}<z_{q}$ if p<q. The displaying sequence is with the convention of left to right, and top to down. For instance, the top left corner one is with $z_{1}$, and the top row second column one is with $z_{2}$. The same applies to the figures hereafter. In the first round, $\beta_{1}=\beta_{2}=6$. In the second round, $\beta_{1}=\beta_{2}=1$.

We reuse the 3-D test in [8], where a coated cube is employed. The cube is with an outer layer ($\epsilon_{r2}=1.5+i0.3$) and an inner layer ($\epsilon_{r1}=2+i0.8$), where the outer edge length is b=2λ and inner edge length is a=1λ, as shown in Figure 4a. In [8], we observe that the inversion with LSIE and FFT-TSOM is able to satisfactorily retrieve the cube while the LSIE with SOM fails. Here, we illustrate how the proposed inversion method with CIE-I and FFT-TSOM performs. Similarly, we carry out two rounds of optimization for this inversion as done in [8] with $M_{F}=6$ and 10, but with CIE-I, we use β=6 in the first round and β=1 for the second round. The reconstruction results are shown in Figure 5, with $ℜ\{{\hat{\epsilon }}_{r}^{\mathrm{est}}\}$ as the real part of the reconstructed relative permittivity and $ℑ\{{\hat{\epsilon }}_{r}^{\mathrm{est}}\}$ as the imaginary part, where we see after two rounds of optimization that the coated cube is successfully found.

**Figure 6:**
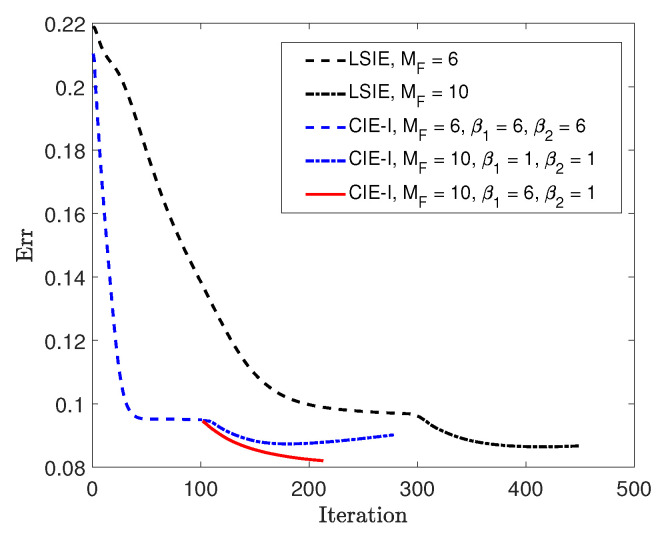
Errors of reconstructions obtained by different inversion methods for *Example 1*.

Now, we compare the convergence speeds of inversions with LSIE and CIE-I. We plot the reconstruction errors for both cases, as shown in Figure 6, in which the inversion with CIE-I appears to be converging much faster than the one with LSIE, with the same reconstruction quality. This confirms again the speeding convergence of inversions in two-dimensional cases shown in [29]. We would like to emphasize that this fast convergence property is extremely important in handling the 3-D ISPs, since the computational costs in 3-D problems are much higher than in two-dimensional cases.

With this example, we further test the relaxed scheme with different β for updating the APCS and the contrast. The termination condition remains the same, $\delta^{3D}<10^{-3}$. We apply this scheme in the second round optimization, with $\beta_{1}=6$ for the APCS update and $\beta_{2}=1$ for the contrast update. For comparison, the recorded reconstruction error at each iteration is plotted in Figure 6 as well, which shows that the convergence is further accelerated compared with the case of using $\beta_{1}=1$.

### 3.2. Example 2

**Figure 7:**
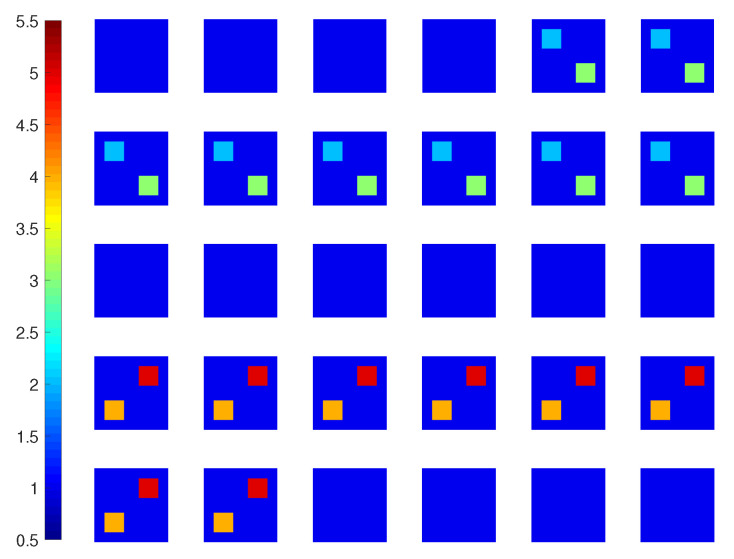
True permittivity profile (real part) for *Example 2*.

In this test, we use a profile with four cubes with increasing contrasts. Having edge length 0.8λ, they are centered at (−0.7,0.7,−0.7)λ, (0.7,−0.7,−0.7)λ, (−0.7,−0.7,0.7)λ, and (0.7,0.7,0.7)λ, with $\epsilon_{r}=2,3,4$ and 5, respectively, as shown in Figure 7.

**Figure 8:**
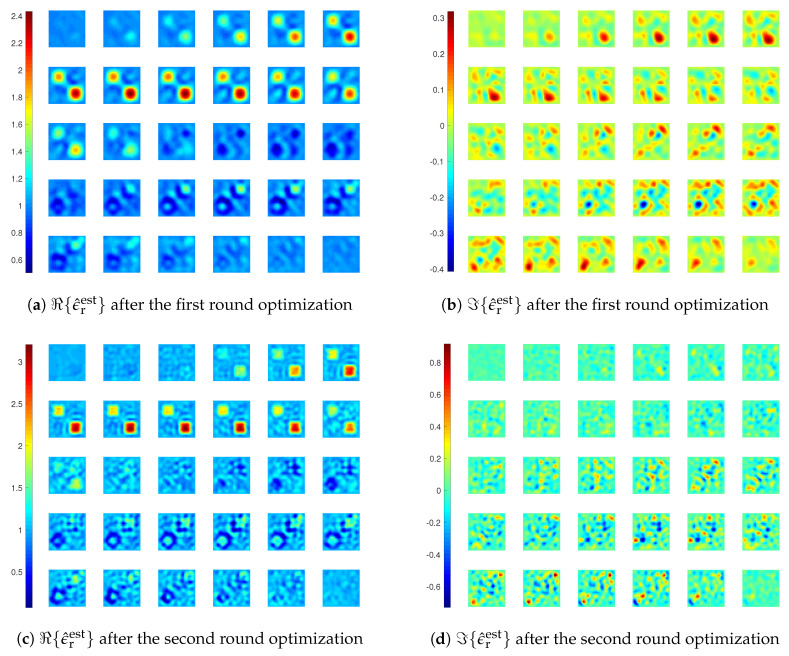
Reconstruction results of inversions with LSIE for *Example 2*.

**Figure 9:**
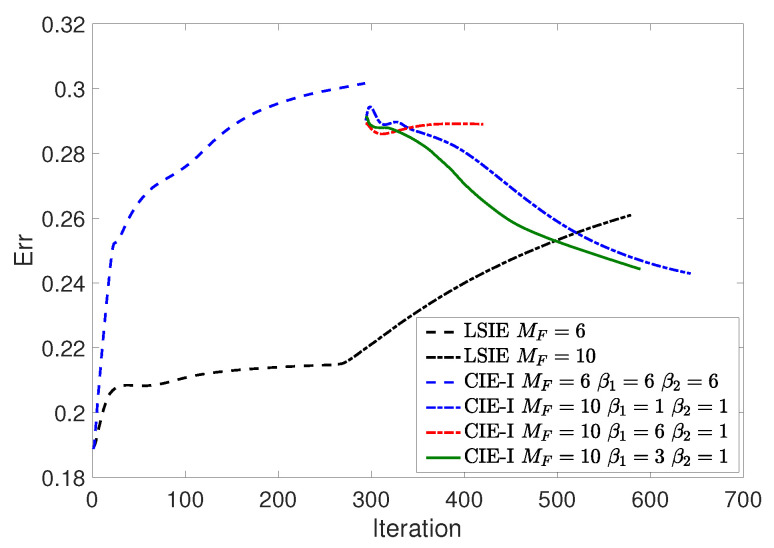
Errors of reconstructions obtained by different inversion methods for *Example 2*.

**Figure 10:**
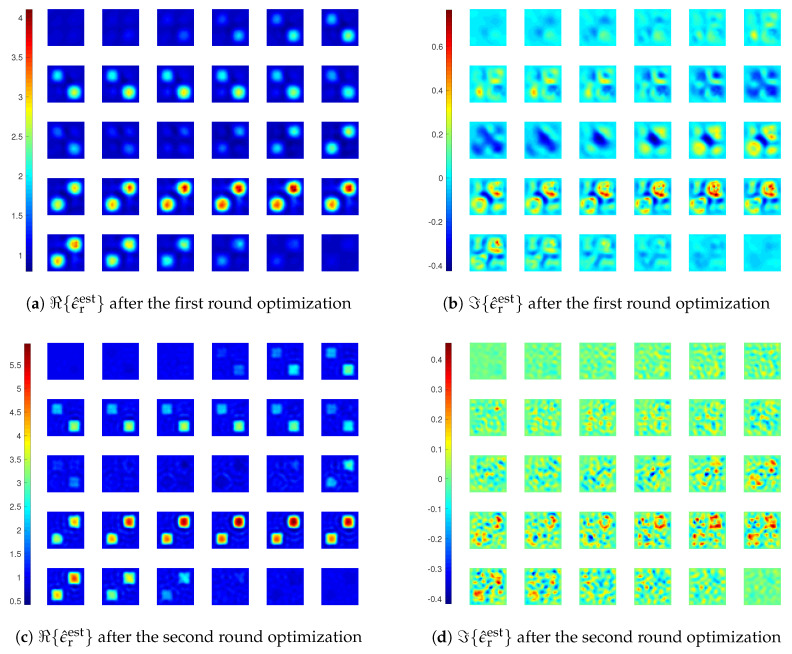
Reconstruction results of inversions with CIE-I for *Example 2*. In the first round, $\beta_{1}=\beta_{2}=6$. In the second round, $\beta_{1}=\beta_{2}=1$.

Two inversions are carried out with LSIE and CIE-I, each with two rounds ($M_{F}=6$ and 10). For CIE-I, the choices of β are 6 and 1 in the two rounds. The reconstruction results and errors are shown in Figure 8, Figure 9 and Figure 10. From these results, we clearly observe that the inversion with LSIE is only able to find the bottom two cubes with lower contrasts, whereas the inversion with CIE-I succeeds in finding all four of four cubes. Reconstruction errors displayed in Figure 9 confirm it, inversion with LSIE diverging while converging with CIE-I.

In addition, we test the inversion scheme with different β for updating the APCS and for updating the contrast. Firstly, we choose $\beta_{1}=6$ for the update of APCS and $\beta_{2}=1$ for the update of the contrast in the second round of optimization with $M_{F}=10$, which however immaturely converges. This might be due to the reason of using a large $\beta_{1}$. Then, we choose $\beta_{1}=3$, and the optimization converges to a good solution with a convergence speed faster than when using $\beta_{1}=1$.

From this example, the inversion with CIE-I is able to handle the highly nonlinear problems consisting of strong scatterers, those with either large contrasts or large dimensions (in terms of wavelength), while the inversion with LSIE may fail.

### 3.3. Example 3

**Figure 11:**
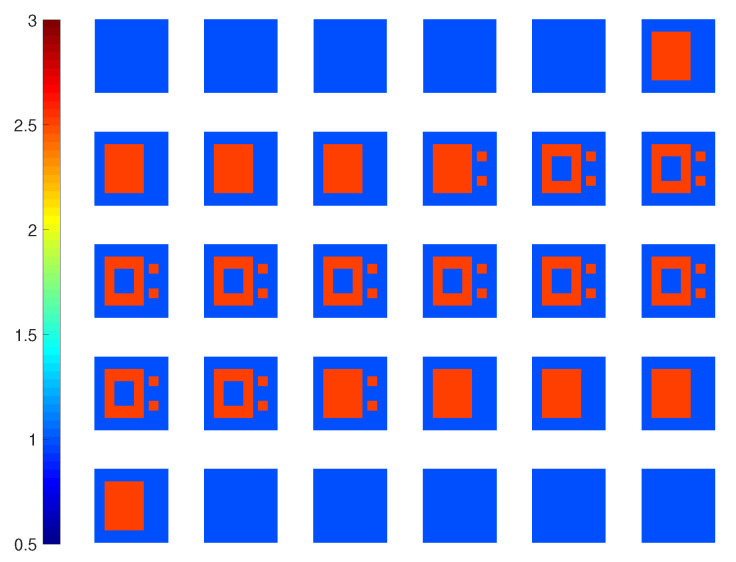
True permittivity profile (real part) for *Example 3*.

We will use another highly nonlinear example to confirm the large difference between the performances of inversion methods with CIE-I and LSIE. In this example, we use a profile that is similar to the famous two-dimensional “Austria” that has an annular and two separated disks. Here, we use a coated cube with a hollow inside and two rods outside the cube, as shown in Figure 11, all of which are with $\epsilon_{r}=2.5$.

**Figure 12:**
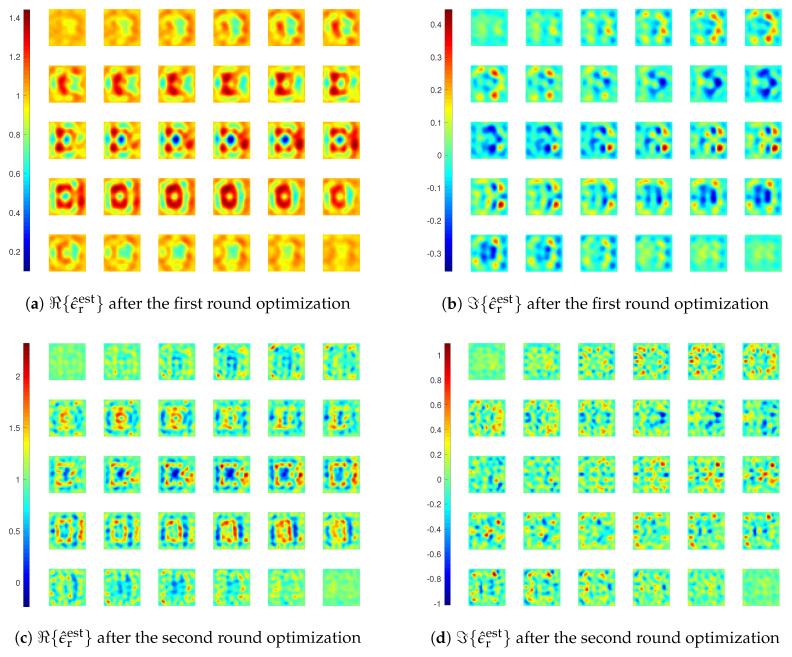
Reconstruction results of inversions with LSIE for *Example 3*.

**Figure 13:**
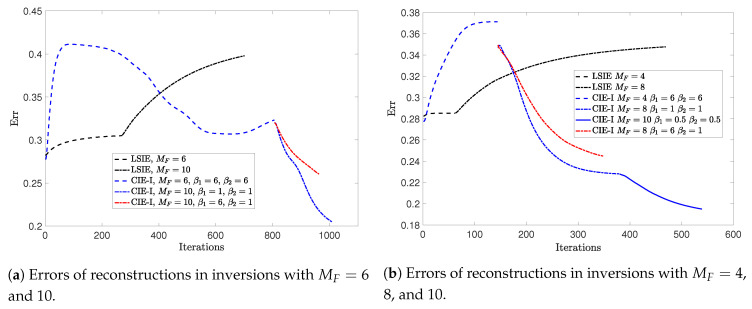
Errors of reconstructions obtained by different inversion methods for *Example 3*.

**Figure 14:**
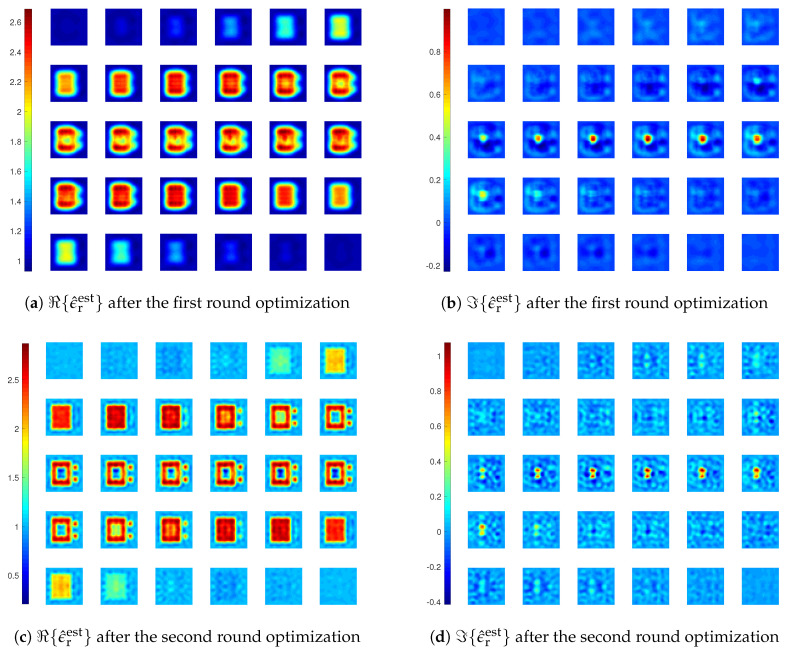
Reconstruction results of inversions with CIE-I for *Example 3*. In the first round, $\beta_{1}=\beta_{2}=6$. In the second round, $\beta_{1}=\beta_{2}=1$.

We still carry out inversions with LSIE and CIE-I with two rounds of optimization, the first with $M_{F}=6$ and the second with $M_{F}=10$. For the CIE-I, $\beta_{1}=\beta_{2}=6$ is used in the first round, and $\beta_{1}=\beta_{2}=1$ in the second round. The reconstructions and errors are shown in Figure 12, Figure 13a and Figure 14. We see that the inversion with LSIE fails to find the profile while the one with CIE-I succeeds. This can also be seen from the errors of the reconstructions in Figure 13a, where the error by the inversion with LSIE diverges.

**Figure 15:**
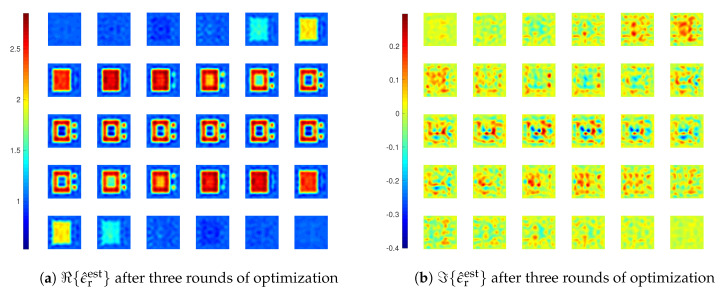
Reconstruction results of inversions with CIE-I after three rounds of optimization for *Example 3*. In the first round, $\beta_{1}=\beta_{2}=6$ and $M_{F}=4$. In the second round, $\beta_{1}=\beta_{2}=1$ and $M_{F}=8$. In the third round, $\beta_{1}=\beta_{2}=0.5$ and $M_{F}=10$.

In the reconstruction results in Figure 14, we see that there is an artefact within the coated cube, where the medium is supposed to be air. This artefact appears in the reconstructed result after the first round optimization with $M_{F}=6$, meaning that the inversion, though capable of retrieving most of the main features of the scatterers, is still not stable enough. Therefore, we carry out another inversion with three rounds of optimization, with $M_{F}=4$, 8, and 10 in each round, and β=6, 1, and 0.5, respectively. The final reconstruction result is given in Figure 15, and we see that there is no more such an artefact.

At last, we also carry out the inversions with different β values for the updates of the APCS and the contrast, and the results are shown in Figure 13. We see that with larger $\beta_{1}$ value for the update of the APCS in the second round optimization, the convergence speed is indeed faster.

From this example, we again confirm the great resolvability of the inversion method with CIE-I against the high nonlinearity of the ISPs.

## 4. Conclusions

In this paper, we propose an inversion method with CIE-I to tackle the 3-D electromagnetic ISPs, especially for highly nonlinear problems with strong scatterers, those with high contrasts or electrically large dimensions. With the CIE-I modeling, the multiple scattering effects in estimating the contrast can be suppressed such that the nonlinearity of inversion can be effectively alleviated. Together with the FFT-TSOM regularization scheme, this largely increases the ability of retrieving the profile of strong scatterers, and effectively accelerates the convergence when handling the moderate scatterers. In addition, by relaxing the contraction mapping, i.e., different contraction mappings are used in updating the contrast sources and in updating the contrast, the convergence of the inversions can be further accelerated. Through numerical examples, we clearly show that the inversions with CIE-I outperform the ones with LSIE, in terms of the resolvability against the nonlinearity and the convergence speed. As shown in [32], the resolvability of the inversion solver with CIE-I against nonlinearity can be further improved in the two-dimensional case if proper regularization techniques are implemented, which should be expected in the 3-D case as well.

## Abbreviations

The following abbreviations are used in this manuscript:

CIE-I	Contraction integral equation for inversion
LSIE	Lippmann–Schwinger integral equation
3-D	Three-dimensional
CSI	Contrast source inversion
SOM	Subspace-based optimization method
FFT-TSOM	FFT type twofold subspace-based optimization method
APCS	Ambiguous part of the contrast source
DPCS	Deterministic part of the contrast source
DoI	Domain of interest
MSE	Multiple scattering effects

## References

[1] Colton D., Kress R. (2013). Inverse Acoustic and Electromagnetic Scattering Theory.

[2] Abubakar A., van den Berg P.M., Mallorqui J. (2002). Imaging of biomedical data using a multiplicative regularized contrast source inversion method. IEEE Trans. Microw. Theory Tech..

[3] Abubakar A., van den Berg P.M. (2000). Three-dimensional inverse scattering applied to cross-well induction sensors. IEEE Trans. Antennas Propag..

[4] Massa A., Boni A., Donelli M. (2005). A classification approach based on SVM for electromagnetic subsurface sensing. IEEE Trans. Antennas Propag..

[5] Sabatier P.C. (2000). Past and future of inverse problems. J. Math. Phys..

[6] Abubakar A., van den Berg P.M. (2004). Iterative forward and inverse algorithms based on domain integral equations for three-dimensional electric and magnetic objects. J. Comput. Phys..

[7] Zhong Y., Chen X., Agarwal K. (2010). An improved subspace-based optimization method and its implementation in solving three-dimensional inverse problems. IEEE Trans. Geosci. Remote Sens..

[8] Zhong Y., Chen X. (2011). An FFT twofold subspace-based optimization method for solving electromagnetic inverse scattering problems. IEEE Trans. Antennas Propag..

[9] Litman A., Lorenzo C. (2009). Special section on testing inversion algorithms against experimental data: 3-D targets. Inverse Probl..

[10] Van den Berg P.M., Kleinman R.E. (1997). A contrast source inversion method. Inverse Probl..

[11] Van den Berg P.M., van Broekhoven A.L., Abubakar A. (1999). Extended constrast source inversion. Inverse Probl..

[12] Wang Y., Chew W.C. (1989). An iterative solution of two-dimensional electromagnetic inverse scattering problem. Int. J. Imaging Syst. Technol..

[13] Chew W.C., Wang Y. (1990). Reconstruction of two-dimensional permittivity distribution using the distorted Born iterative method. IEEE Trans. Med. Imaging.

[14] Dorn O., Lesselier D. (2006). Level set methods for inverse scattering. Inverse Probl..

[15] Benedetti M., Lesselier D., Lambert M., Massa A. (2009). A multi-resolution technique based on shape optimization for the reconstruction of homogeneous dielectric objects. Inverse Probl..

[16] Chen X. (2010). Subspace-based optimization method for solving inverse scattering problems. IEEE Trans. Geosci. Remote Sens..

[17] Zhong Y., Chen X. (2009). Twofold subspace-based optimization method for solving inverse scattering problems. Inverse Probl..

[18] Agarwal K., Pan L., Chen X. (2009). Subspace-based optimization method for reconstruction of two-dimensional complex anisotropic dielectric objects. IEEE Trans. Microw. Theory Tech..

[19] Pastorino M. (2007). Stochastic optimization methods applied to microwave imaging: A review. IEEE Trans. Antenna Propag..

[20] Rocca P., Benedetti M., Donelli M., Franceschini D., Massa A. (2009). Evolutionary optimization as applied to inverse scattering problems. Inverse Probl..

[21] De Zaeytijd J., Franchois A., Geffrin J.M. (2009). A new value picking regularization strategy-Application to the 3-D electromagnetic inverse scattering problem. IEEE Trans. Antenna Propag..

[22] Chaumet P., Belkebir K. (2009). Three-dimensional reconstruction from real data using a conjugate gradient-coupled dipole method. Inverse Probl..

[23] Yu C., Yuan M., Liu Q.H. (2009). Reconstruction of 3-D objects from multi-frequency experimental data with a fast DBIM-BCGS method. Inverse Probl..

[24] Donelli M., Franceschini D., Rocca P., Massa A. (2009). Three-dimensional microwave imaging problems solved through an efficient multiscaling particle swarm optimization. IEEE Trans. Geosci. Remote Sens..

[25] Agarwal K., Chen X., Zhong Y. (2010). A multipole-expansion based linear sampling method for solving inverse scattering problems. Opt. Express.

[26] Bevacqua M.T., Isernia T. (2018). Boundary Indicator for Aspect Limited Sensing of Hidden Dielectric Objects. IEEE Geosci. Remote Sens. Lett..

[27] Isernia T., Crocco L., D’Urso M. (2004). New tools and series for forward and inverse scattering problems in lossy media. IEEE Geosci. Remote Sens. Lett..

[28] D’Urso M., Isernia T., Morabito A.F. (2010). On the Solution of 2-D Inverse Scattering Problems via Source-Type Integral Equations. IEEE Trans. Geosci. Remote Sens..

[29] Zhong Y., Lambert M., Lesselier D., Chen X. (2016). A new integral equation method to solve highly nonlinear inverse scattering problems. IEEE Trans. Antennas Propag..

[30] Pankratov O.V., Avdeyev D.B., Kuvshinov A.V. (1995). Electromagnetic field scattering in a heterogeneous earth: A solution to the forward problem. Phys. Solid Earth.

[31] Peterson A.F., Ray S.L., Mittra R. (1998). Computational Methods for Electromagnetics.

[32] Xu K., Zhong Y., Wang G. (2018). A hybrid regularization technique for solving highly nonlinear inverse scattering problems. IEEE Trans. Microw. Theory Tech..

